# The evolutionary history of cribellate orb-weaver capture thread spidroins

**DOI:** 10.1186/s12862-022-02042-5

**Published:** 2022-07-09

**Authors:** Sandra M. Correa-Garhwal, Richard H. Baker, Thomas H. Clarke, Nadia A. Ayoub, Cheryl Y. Hayashi

**Affiliations:** 1 Division of Invertebrate Zoology and Sackler Institute for Comparative Genomics, American Museum of Natural History, New York, NY USA; 2 Department of Biology, Washington and Lee University, Lexington, VA USA

**Keywords:** Cribellate silk, Spidroin, Spider silk, Evolution, Gene family, Genomics, Transcriptomics

## Abstract

**Background:**

Spiders have evolved two types of sticky capture threads: one with wet adhesive spun by ecribellate orb-weavers and another with dry adhesive spun by cribellate spiders. The evolutionary history of cribellate capture threads is especially poorly understood. Here, we use genomic approaches to catalog the spider-specific silk gene family (spidroins) for the cribellate orb-weaver *Uloborus diversus*.

**Results:**

We show that the cribellar spidroin, which forms the puffy fibrils of cribellate threads, has three distinct repeat units, one of which is conserved across cribellate taxa separated by ~ 250 Mya. We also propose candidates for a new silk type, paracribellar spidroins, which connect the puffy fibrils to pseudoflagelliform support lines. Moreover, we describe the complete repeat architecture for the pseudoflagelliform spidroin (Pflag), which contributes to extensibility of pseudoflagelliform axial fibers.

**Conclusions:**

Our finding that Pflag is closely related to Flag, supports homology of the support lines of cribellate and ecribellate capture threads. It further suggests an evolutionary phase following gene duplication, in which both Flag and Pflag were incorporated into the axial lines, with subsequent loss of Flag in uloborids, and increase in expression of Flag in ecribellate orb-weavers, explaining the distinct mechanical properties of the axial lines of these two groups.

**Supplementary Information:**

The online version contains supplementary material available at 10.1186/s12862-022-02042-5.

## Background

Spiders are known for making a variety of task-specific silk fibers with diverse material properties [1]. The evolutionary success of spiders can be attributed to their wide ranging use of silk. For example, orb-web weavers use multiple silks in web construction, with each type synthesized in a morphologically specialized silk gland. The combination of different silk types makes the orb-web a strong yet extensible prey-catching system [2, 3]. The web frame and radii are primarily made of major ampullate silk, the attachment disks are made of pyriform silk, and the capture spiral is a composite of a stretchy filament and sticky silks. There are two types of capture spirals. One type has a pair of proteinaceous fibers produced by flagelliform glands that are coated with moist, adhesive droplets produced by aggregate glands [4]. This type of capture spiral is spun by over 4,600 species of ecribellate spiders in seven spider families (superfamily Araneoidea), the largest and most commonly encountered being the family Araneidae [5]. The protein adhesive in araneid capture lines is also found in the gumfoot lines of cobwebs which are constructed by the ~ 2,800 species of Theridiidae and Nesticidae [6, 7]. In contrast, cribellate capture threads are supported by a pair of pseudoflagelliform fibers, which are covered with tangles of dry, mechanically sticky cribellate nanofibrils [8–11]. Cribellate threads also are spun by various basal members of the infraorder Araneomorphae, which encompasses the largest number of living spider species. However, members of only 21 of the 96 families in Araneomorphae continue to spin cribellate threads [12]. Among orb-web building spiders, the transition from dry cribellate to viscous sticky capture threads is not well understood and whether cribellate and ecribellate webs evolved in parallel [13–18] or cribellate silk was replaced by sticky lines [19, 20] is still uncertain.

Among the cribellate spiders are members of the family Uloboridae. These small to medium-sized spiders are one of only two spider families to lack venom glands [21, 22]. The family Uloboridae currently encompasses 19 genera and 287 species distributed worldwide [5]. Uloborids are known to have a laborious prey wrapping technique that results in tight swaddling, thought to be a way of compensating for the lack of venom to immobilize prey [10, 23–25]. Most uloborid species construct fully circular orb-webs while other species construct reduced orb-webs, such as the triangle-webs of *Hyptiotes* or the single-line capture threads of *Miagrammopes* [10, 26].

Cribellate silk is produced by a very complex and specific spinning process; thousands of ultrafine fibrils are spun simultaneously from a plate-like spinning organ with numerous spigots, called the cribellum, onto an extensible core fiber of pseudoflagelliform silk. The fibrils are vigorously combed into woolly snarls by a row of specialized leg bristles—the calamistrum [27–29]. In the uloborid capture spiral, the cribellar fibrils surround the supporting axial fibers [8, 9, 30]. The capture spiral also possesses connecting fibers produced in paracribellate glands [31, 32]. Non-uloborid cribellate species can have a more complex cribellate silk that includes highly folded reserve warp fibers (also called undulating fibers) that are likely produced in minor ampullate glands [4, 30, 33–35]. Cribellate capture threads can stick to many different surfaces and they achieve this adhesion via a combination of hygroscopic forces, van der Waals’ forces, entanglement of cuticular structures, and the absorption of cuticular waxes from prey [36–39]. The mechanical properties of capture thread are influenced by the use of different fibers, making cribellate silk both stiff and extensible. While the pseudoflagelliform axial fibers provide stiffness, strength, and initial elasticity, the irreversible unfolding of cribellar fibrils allow the capture thread to extend as much as 500% relative to its original length [40].

The mechanical behavior of spider silk has also been attributed to its protein composition. Spider silks are mainly composed of silk proteins called spidroins (a contraction of spider fibroin; [41]). Spidroins are encoded by a single gene family and are highly expressed in silk glands [42–49]. While silk proteins for most silk types have been described, the molecular composition of cribellate threads has only recently gained attention. Sequences from several cribellate spiders show that cribellar fibrils and pseudoflagelliform fibers are composed of distinct spidroins specific to cribellate and pseudoflagelliform glands, respectively [49, 50]. Yet, detailed examination of the genetics of cribellate silk and how particular genes relate to the evolution of cribellate silk components is not well understood. For instance, candidates for the paracribellar spidroins, the main components of paracribellar connecting fibers, have not been identified. Here, we describe a comprehensive spidroin set for the uloborid orb-weaver *Uloborus diversus* (Walckenaer, 1841) using genomic and transcriptomic data and examine, in detail, the spidroins associated with the three types of silk in cribellate capture threads: cribellar fibrils, pseudoflagelliform fibers, and paracribellar fibers. We present the complete sequence of pseudoflagelliform spidroin and identify potential spidroin candidates for paracribellar silk. We also show that pseudoflagelliform spidroins are closely related to the flagelliform spidroins, suggesting a shared origin of the two fiber types.

## Results

### *Uloborus diversus* genome and transcriptome assembly

We obtained a total of 1571 billion reads from the genome sequencing of *U. diversus*. These reads were assembled using Supernova 2.0.1 (10X Genomics) and the resulting genome assembly was 1.49 Gb and included 47,680 scaffolds greater than 10 kb (scaffold N50: 42.36 kb). Based on published flow cytometry data, one *Uloborus* species has a genome size of approximately 2.5 Gb [51], suggesting our assembly is incomplete. Nevertheless, the assembled genome was found to have 90% of the Benchmarking Universal Single-Copy Orthologs (BUSCO [52]) genes as either complete (65%) or fragmented (25%). In total, 17,878 high quality Iso-Seq transcripts were obtained with a mean length of 3980.6 bp. For the RNA-Seq assembly, an average of 23.5 and 23.2 million cleaned reads were obtained from the two total silk libraries and two cephalothorax libraries, respectively.

The *de novo* assembly of the RNA-Seq reads had 542,127 transcripts with an N50 of 794. Since Trinity transcriptomes can contain both multiple alleles per gene and potentially low-quality transcripts, we developed a novel trimming and annotation pipeline called TrTAP to reduce the *de novo* transcriptome to a set of high confidence genes (Additional file 3: Figure S1). The pipeline begins by comparing the transcripts to multiple gene-specific databases (here spidroins) or to high-quality complete gene sets from related species using BLAST. The pipeline then parses the BLAST results to select one representative allele per Trinity cluster as well as exclude chimeric and RNA transcripts. For the clusters where there are no BLAST matches, the longest ORF is used in the selection, with such ORFs greater than 50 amino acids. The pipeline annotates all allele clusters using comparisons to PFAM and SwissProt and calculates their expression abundance. In the final step, the pipeline removes transcripts that are not best hits to the gene databases and have low coverage of matching genes or low expression using RSEM Transcripts per Million (TPM). The resulting pruned transcriptome is composed of 115,961 proteins, of which 26,088 had homologous mapping to known genes. This pruned set contains a majority of the expression and annotated genes as well as differentially expressed genes (Additional file 2: Table S1). Single copy orthologs analysis, BUSCO [52], indicates that the trimmed transcriptome is 91% complete. The pruned set has matches to 87.4% of the BUSCO genes. The missing 3.6% BUSCO genes not found in the pruned set matched to genes removed for having low expression values.

### Spidroin complement and expression in *U. diversus*

**Fig. 1:**
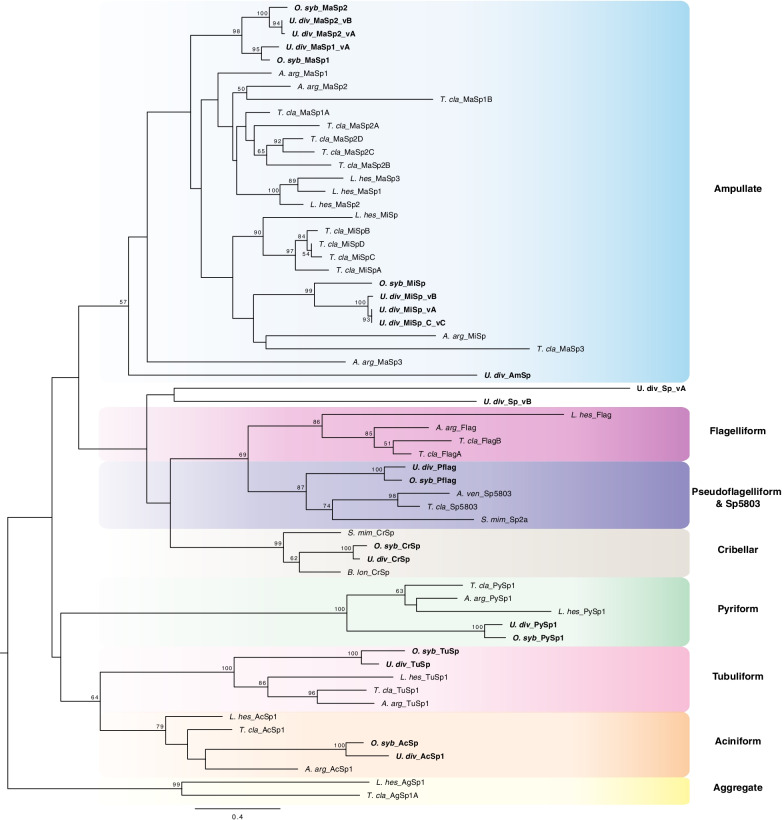
Spidroin gene tree (maximum likelihood) of concatenated N- and C- terminal region protein sequences. Shaded rectangles indicate spidroin types, annotated as ampullate (blue), flagelliform (magenta), pseudoflagelliform and Sp5803 (violet), cribellar (brown), pyriform (green), tubuliform (pink), aciniform (orange), and aggregate (yellow). Tree rooted with California trapdoor spider *Bothriocyrtum californicum* fibroin 1 (not shown). See Additional file 3: Table S2 for spidroin sequence information. Bootstrap percentages ≥ 50% are shown. Scale bar represents replacements per site. Uloboridae spidroins are highlighted in bold

We identified 18 spidroin genes expressed in *U. diversus* silk glands. For some spidroin genes, full length nucleotide sequences were obtained from single 10X contigs, for those that were not, reconstruction was done using a combination of 10X contigs and Iso-Seq reads. Full or partial length spidroin genes were assembled for all expected spidroin types. Aciniform (AcSp), tubuliform (TuSp), minor ampullate (MiSp), major ampullate (MaSp), pseudoflagelliform (Pflag) spidroins, and two spidroins that could not be assigned to a known spidroin type, annotated with the general name Spidroin and a version letter (Sp_vA and Sp_vB), all appear to be complete, full-length genes. The *U. diversus* ampullate spidroin (*U. div*_AmSp) also appears to be full-length but could not be categorized as either MaSp or MiSp. The region bounded by the terminal domains of *U. div*_AmSp is not repetitive and lacks the characteristic amino acid motifs of MaSp or MiSp, but the terminal regions share conserved spidroin amino acid signatures (Additional file 3: Figure S2). The N- terminal region has the conserved amino acid motifs found to be restricted to MaSp spidroins (e.g. amino acid motifs PW, AxxFxxxxF from [53]) and the C-terminal region has the charged amino acid residues that form salt bridges and aid in monomer stabilization on MaSp and MiSp spidroins [53–55]. We recovered all of the previously published partial spidroin sequences for *U. diversus* by Garb and colleagues [56, 57]. Spidroin annotation was based on spidroin-specific gene characteristics, such as the presence of spidroin-specific motifs in the terminal domains (see Collin et al. [53]) and repeat composition. Since spidroins are members of a gene family, annotation was also supported by evolutionary relationship information from spidroin gene trees (Fig. 1).

The set of spidroin genes in *U. diversus* is consistent with the set recently described for six species in the uloborid genus *Octonoba* by Kono et al. [50]. The amino acid composition, approximate length, and organization of *U. diversus* spidroins are consistent not only with those of *Octonoba* but also with the spidroins of non-uloborid spiders [49, 57–59]. For example, the complete sequence of AcSp1 is composed of ten tandem repeats that are on average 357 amino acids long and account for ~ 91% of the protein (Additional file 3: Figure S2). Like previously described AcSp sequences, *U. diversus* AcSp1 is enriched in the amino acids serine (25%), alanine (14%), and glycine (8%). Phylogenetic analyses of N- and C- terminal coding regions show *Uloborus* and *Octonoba* spidroin sequences cluster together with high bootstrap support (e.g. ≥ 99%) within clades of the same type of spidroin from other species (e.g., *U. div* and *O. syb* AcSp1 in an AcSp1 clade; Fig. 1 orange box).

Confirmation of *U. diversus* spidroin functionality was obtained from expression analyses using transcripts derived from total silk gland and cephalothorax tissues (Additional file 3: Figure S3). We found that all identified spidroin genes are expressed in silk glands (relative to the cephalothorax; DESeq analysis, padj < 0.001), most at high levels (Additional file 3: Figure S3). Moreover, the type, number, and expression of spidroins in *U. diversus* is similar to the silk genes expressed in *Octonoba* species [50]. Aside from spidroin genes, there are 1,357 genes that are up-upregulated in uloborid silk glands compared to cephalothorax. The functions of these proteins suggest they are involved in oxidoreductase and transmembrane transport, similar to what has been described in theridiid and araneid species [48, 60, 61]. Given the functional similarities of the dry adhesive used by cribellate spiders and the wet glue in ecribellate orb-web and cobweb weavers, we searched for genes in *U. diversus* genome with sequence similarly to Theridiidae aggregate gland specific OESTs (Over Expressed Sequence Transcripts [48]). These aggregate OESTs are thought to be important in the production of aggregate glue droplets. We found 21 *U. diversus* genes with a match to 11 OESTs (e-values ≤ e−50). However, mapping RNA-seq reads to these 21 genes shows that they are lowly expressed in both total silk and cephalothorax tissues (Additional file 3: Table S2). These results suggest that the *U. diversus* genome has homologs to only a fraction of the of cobweb weaver aggregate-specific OESTs (> 300 per species), and that these do not have similar roles to cobweb weaver proteins.

**Cribellate capture thread spidroins in Uloboridae**

**Fig. 2:**
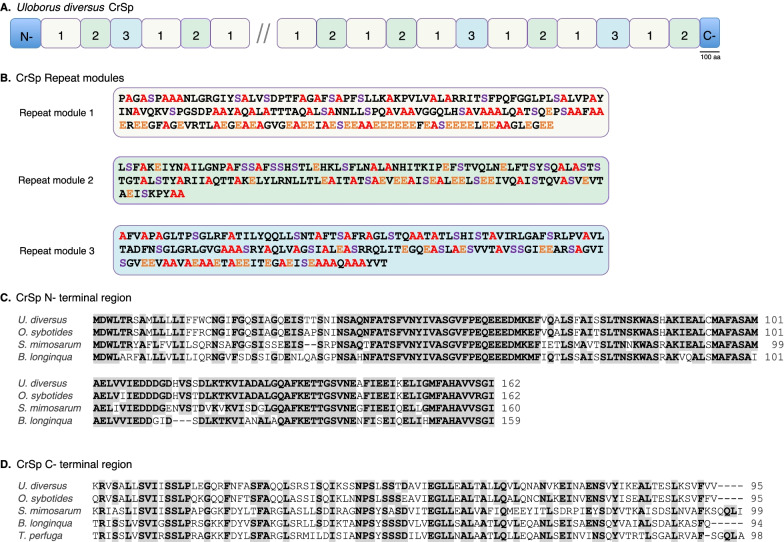
Cribellar spidroin (CrSp) structure of *Uloborus diversus*. **A** Schematic of CrSp repeat region organization and its three repeat types. Conserved spidroin amino and carboxyl terminal region are shown as N- and C-, respectively (blue boxes). Double forward slashes indicate missing data. Numbered boxes represent repeat module types. Scale bar indicates 100 amino acids. **B** Consensus sequences of the three repeat module types indicated by single-letter amino acid abbreviations. Abundant CrSp amino acids highlighted as follows: alanine (red), serine (purple), glutamic acid (orange). Multiple sequence alignment of CrSp conserved **C** N-terminal and **D** C-terminal regions of the cribellate species *U. diversus*, *Octonoba sybotides*, *Stegodyphus mimosarum*, *Badumna longinqua*, and *Tengella perfuga*. See Additional file 3: Tables S3 and S4 for name and sequence information. Bold and grey shaded amino acids are conserved > 75% across species. Total amino acid number shown on the right. Dashes indicate alignment gaps

Uloborid capture threads are composed of three different silk types, thus we expected to find genes corresponding to those silks: cribellar spidroin (CrSp), pseudoflagelliform spidroin (Pflag), and at least one spidroin candidate for paracribellar silk, which has yet to be identified in any species [49, 50]. Indeed, we found gene sequences that match the cribellar spidroin CrSp (Fig. 2). The repetitive region of *U. diversus* CrSp was found to have three distinct types of repeat modules (Fig. 2B). Each module type has a distinctive amino acid combination and is repeated multiple times within the repetitive region of *U. diversus* CrSp (Fig. 2A, B). These repeat modules are not unique to *U. diversus*, they are also present in CrSp orthologs from other cribellate spiders such as *Octonoba*, *Tengella*, *Stegodyphus*, and *Badumna* [49, 50, 59]. For example, repeat module 1 has a 79% amino acid (aa) identity within Uloboridae and a range of 28–34% aa identity between uloborids and other families, suggesting it has been conserved for at least ~ 250 Mya (Additional file 3: Figure S4) [17, 62]. Repeat modules 2 and 3 do not align well outside of Uloboridae while having 75% and 69% identity within Uloboridae, respectively (Additional file 3: Figure S4). Comparison of CrSp repeat structure across species shows that unlike previously reported by Kono et al. [50], *Octonoba* CrSp is also composed of three repeat units that are homologous to those of *U. diversus* CrSp but differ significantly at the higher level repeat arrangement (Additional file 3: Figure S4A). The terminal regions of cribellar spidroins show conservation across species, with N- and C-terminal regions sharing 56% and 76% identity at the aa level, respectively (Fig. 2C, D). Phylogenetic analysis further grouped CrSp sequences into a well-supported monophyletic clade (Fig. 1 and 99% BT).

**Fig. 3:**
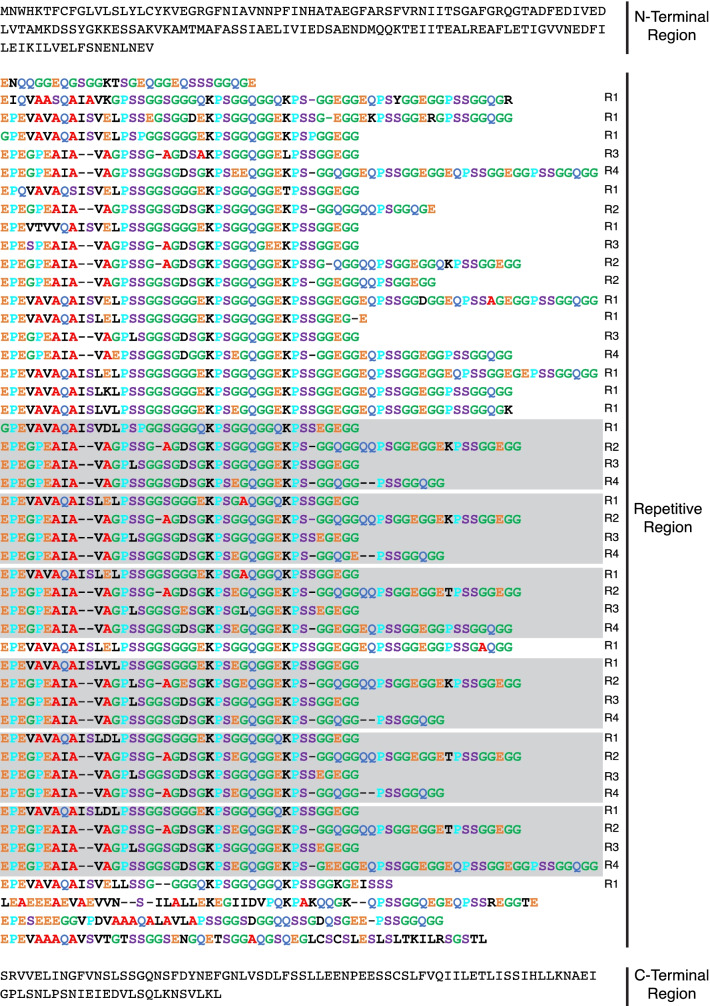
Amino acid sequence of the complete *Uloborus diversus* pseudoflagelliform spidroin, showing the N-terminal, repetitive, and C-terminal regions. Abundant amino acids are highlighted as follows: alanine (red), serine (purple), glycine (green), glutamine (blue), proline (cyan), and glutamic acid (orange). Each ensemble repeat is labeled on the right by their repeat type classification (see Additional file 3: Figure S2). Recurring, higher level pattern of repeats (R1, R2, R3, R4) are indicated by grey boxes

The full-length *U. diversus Pflag* gene is approximately 8 kbp in size and codes for an ~ 2600 aa long protein (Fig. 3). The repetitive sequence of Pflag has 48 ensemble repeats that cluster into four distinct repeat types (Additional file 3: Figure S5). Each repeat has an 11–13 aa spacer motif, which is a highly conserved glycine-poor region that interrupts glycine-rich motifs [43], followed by variations of the aa motif XPSSGGXGGXEK, where X can be the amino acids: G, S, A, Q, E, or D (Fig. 3). These repeats are further organized in recurring 4-mer repeats (Fig. 3). Comparative analysis of the repeat structure of Pflag led us to define multiple ensemble repeats in *Octonoba* Pflag, which is different than the single unit described by Kono et al. [50] (Additional file 3: Figure S6). The sequence similarity among the multiple ensemble repeats in *Octonoba* Pflag is not as strongly correlated as those of CrSp, yet it shows a 4-mer repeat in the higher repeat structure (Additional file 3: Figure S6). Unlike the flagelliform spidroin gene (*Flag*) from ecribellate orb-web weavers [63–65], uloborid *Pflag* does not appear to have introns. Furthermore, we found Pflag sequences to have a lower percentage of glycine and proline amino acids (G: ~38%, P: ~11% for *U. diversus* and *O. sybotides*) compared to Flag sequences (G: ~50%, P: ~14% for *A. ventricosus* and *T. clavipes*). Moreover, Pflag amino acid motifs are not the same as in Flag; instead Pflag has the proline-containing motifs GPS(X), KPS(X), and QPS(X), with KPS(X) being the dominant motif in *U. diversus* Pflag (Fig. 3).

**Fig. 4:**
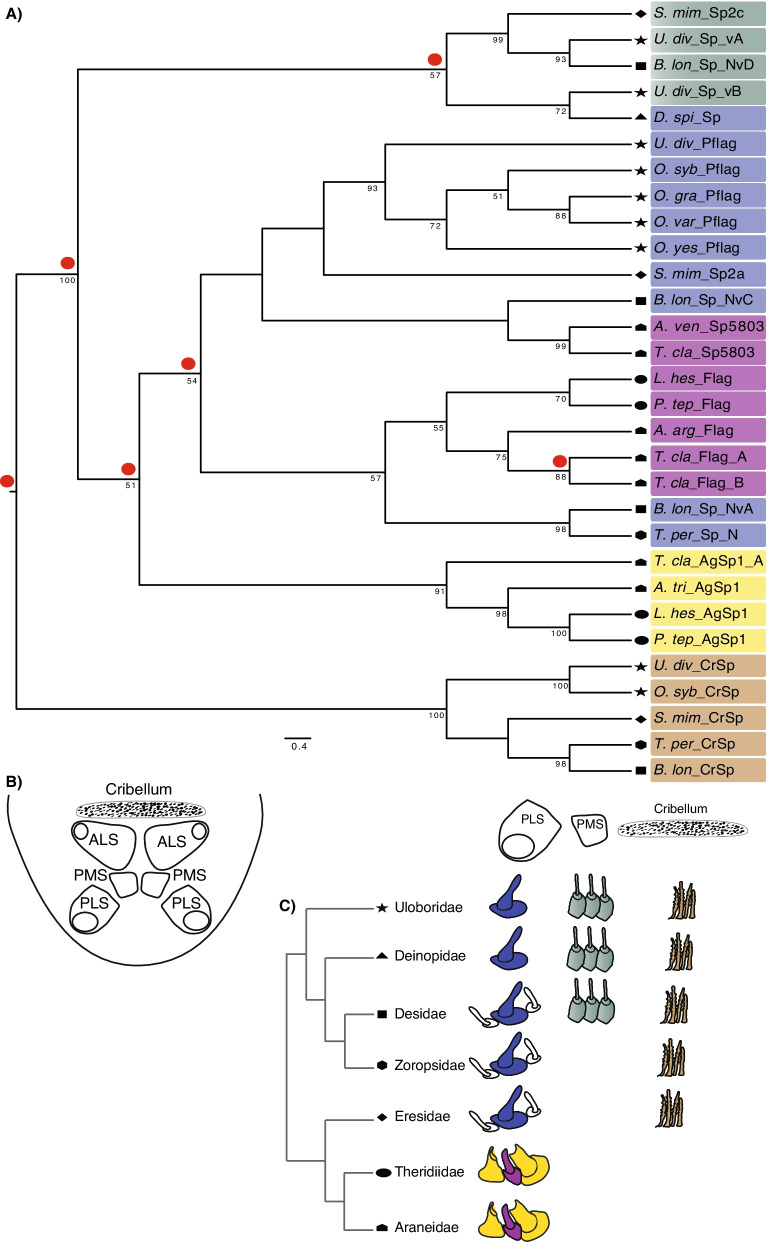
**A** Maximum likelihood analysis of concatenated nucleotide N- and C- terminal regions of spidroins involved in capture thread construction. Tree is rooted on cribellar spidroins. Species names are abbreviated as in Fig. 1. Bootstrap percentages (BT) ≥ 50% are shown. Each spidroin is colored based on the silk gland where it is likely expressed: flagelliform (purple), pseudoflagelliform (blue), aggregate (yellow), cribellar (brown), paracribellar (green). Inferred gene duplications are shown as red circles above each branch for those supported by > 50% BT. Symbols show familial association as follow: Uloboridae (star), Deinopidae (triangle), Desidae (square), Zoropsidae (hexagon), Eresidae (diamond), Theridiidae (oval), and Araneidae (pentagon). **B** Schematic of the spinnerets of a cribellate spider showing the cribellum, the anterior lateral spinnerets (ALS), posterior median spinnerets (PMS), and posterior lateral spinnerets (PLS). **C** Spigot morphology information for silks involved in capture thread construction showing on which spinneret the spigots are located. Spigots are colored as in part A: flagelliform spigot (purple), pseudoflagelliform/modified spigot (blue), aggregate spigots (yellow), cribellar spigots (brown), and paracribellar spigots (green). See Additional file 3: Table S4 for spidroin sequence information. Spider phylogeny is based on [67]

The Pflag N-terminal domain is most closely related to Sp5803 (Fig. 1), a spidroin that is expressed in flagelliform silk glands (but is not Flag) and that also lacks introns [66]. The spidroin Sp5803, described in the orb-web weaving *Trichonephila clavipes*, has a unique repetitive region relative to all other described spidroin types and lacks the conserved spidroin C-terminal region [64, 66]. BLAST searches to published spider genomes, revealed the presence of Sp5803 in *Araneus ventricosus* (Accession GBL75419.1) based on the presence of the conserved spidroin N-terminal region, similar repetitive region to *T. clavipes* Sp5803, and absence of conserved spidroin C-terminal region. Phylogenetic analyses show that *U. diversus* Pflag is in a clade with Pflag spidroins from other cribellate spiders (Figs. 1, 4). Moreover, we found phylogenetic evidence for ecribellate orb-web weaving Flag spidroins to share a most recent common ancestor with Pflag/Sp5803 (69% BT), which together are closely related to CrSp (Figs. 1, 4).

The molecular components of paracribellar silk are unknown. We propose two candidate paracribellar spidroins: *U. div* Sp_vA and *U. div* Sp_vB. These spidroins appear to be full-length and have divergent repetitive sequence from each other and other spidroin types. While *U. div*_Sp_vA and *U. div*_Sp_vB possess the conserved spidroin terminal domains that include positionally conserved amino acid motifs [53], they did not group with well-established silk gene types in our phylogenetic analysis, suggesting they may represent new spidroin types. The repetitive regions of *U. div*_Sp_vA and *U. div*_Sp_vB, like known spidroins, are organized into tandemly arranged repeat units. *U. div*_Sp_vA has eight repeat units that are highly similar to each other with 86% identity at the aa level, and the repeats are enriched for the amino acids alanine (21%), serine (16%), glycine (11%) and valine (10%) (Additional file 3: Figure S2). *U. div*_Sp_vB repeat region is rich in serine (17%) and alanine (12%) amino acids; it has a 28 aa repeat module that is repeated five times, followed by a region with no recognizable modules (Additional file 3: Figure S2). Considering overall amino acid composition, *U. div*_Sp_vA and *U. div*_Sp_vB are similar to tubuliform and aciniform spidroins, but the repeat modules do not align well to these other paralog groups.

We examined spigot morphological data within a phylogenetic framework to investigate how capture thread spidroins are associated with evolutionary changes in spigot types. Gene family analyses of concatenated N- and C- terminal nucleotide sequences of spidroins genes involved or thought to be involved in capture thread production, show that *U. div*_Sp_vA and *U. div*_Sp_vB cluster within a clade with other “Sp”. Other spidroins in that clade also have divergent repetitive regions and have not been assigned a spidroin type (Fig. 4). Unexpectedly, *D. spinosa* spidroin grouped with *U. div*_Sp_vB, likely a result of *D. spi*_Sp sequence lacking an N-terminal region and having a divergent C-terminal region that has similarities to that of *U. div*_Sp_vB. Moreover, unlike *U. div*_Sp_vB, the repetitive region of *D. spi*_Sp is like other Pflag and Flag sequences [57]. Two transcripts containing the N-terminal region only, *B. lon*_Sp_NvA and *T. per*_Sp_N grouped together as a sister clade of Flag (Figs. 4 and 57% BT), a grouping consistent with previous spidroin gene tree analysis [59]. The available repetitive regions of *T. per*_Sp_N and *B. lon*_Sp_NvA are short, enriched in the amino acids glycine (22% and 23% for *T. per*_Sp_N and *B. lon*_Sp_NvA, respectively), glutamic acid (10% and 11%), and serine (9% and 10%), and lack amino acid motifs corresponding to Flag or Pflag spidroins.

## Discussion

Cribellate silk is spun by thousands of araneomorph spider species with a wide ecological and taxonomic distribution [68, 69]. Our understanding of spider silks and silk genetics is largely based on ecribellate orb-web weavers, leaving cribellate silk genetics relatively unstudied. Our identification and annotation of the silk gene catalog of *U. diversus* greatly increases our knowledge of spidroin diversity in cribellate species and sheds light into the evolution of cribellate silk genes. Sequence similarity and phylogenetic affinity of *U. diversus* spidroins to those of *Octonoba* species suggest that the ancestor of these uloborids had a similar complement of silk proteins likely dating back to ~ 145 Mya [70]. Spidroin sequences not related to the capture threads (e.g. AcSp, TuSp, PySp) show conservation in their terminal regions, as well as in repetitive regions, with previously described sequences [45, 49, 50, 64, 71–74]. This sequence conservation suggests similar selective pressures have acted on these spidroins in diverse spider lineages. Uloborid ampullate sequences form monophyletic clades with moderate support (*U. div* + *O. syb* MaSp1, MaSp2, and MiSp; Fig. 1), within a large, diverse MaSp and MiSp spidroin clade (Fig. 1). The grouping of uloborid MaSp1 and MaSp2 to the exclusion of MaSp1 and MaSp2 of araneoid spiders suggests gene conversion of the terminal domains in the ancestor of uloborids, similar to what has been proposed for black widows [75]. It is also possible that extensive convergent evolution of MaSp1-like and MaSp2-like repetitive regions has occurred in divergent orb-web weaver clades.

Since cribellate silk production is ancestral for the hyper-diverse infraorder Araneomorphae, a fundamental knowledge of the molecular constituents of cribellate capture thread is pivotal to our understanding of spider silk evolution. Uloborid cribellate silk is a composite of multiple fiber types that are produced in distinct silk glands: the core axial fibers are produced in a pair of pseudoflagelliform silk glands, cribellar nanofibers that form the “puffs” of cribellate silk are produced in several thousand cribellar silk glands, and paracribellar silk, which connects nanofibers to the axial fiber, come from ~ 20 paracribellate silk glands [8, 10, 11, 76]. The combination of these fibers gives cribellate silk its woolly yarn appearance, adhesiveness, and mechanical properties [37–40, 77–79].

Flagelliform silk is the main component of the core axial fibers of capture threads in ecribellate spiders and is produced in flagelliform silk glands [43]. Pseudoflagelliform silk of cribellate orb-web weaving spiders is thought to be homologous to flagelliform silk of ecribellate orb-web and cob-web weaving spiders because the spigots that produce these fibers are located on the same pair of spinnerets in both groups. That is, they have positional homology [19, 30, 80, 81]. It follows that the glands associated with those spigots are also homologous. Furthermore, under the glandular affiliation hypothesis, we would expect proteins to have evolved in association with the silk gland where they are mainly expressed [43]. Gene tree analyses show that spidroins associated with core axial fibers are within a clade of Flag and Pflag plus Sp5803 spidroins (Figs. 1 and 4). The well-supported grouping of uloborid Pflag spidroins with Sp5803 and *S. mimosarum Sp2a* suggests these latter spidroins are, in fact Pflag orthologs. We also propose that the *D. spinosa* spidroin (ABD61590) described by Garb et al. [57] is likely to be expressed in pseudoflagelliform silk glands, but is neither a Pflag ortholog nor a Flag spidroin as previously thought. In terms of Flag and Pflag evolution, we posit that these two spidroin types are the results of a duplication event in the common ancestor of araneoids, uloborids, the RTA-clade, and eresids (Entelegynae), approximately 250 Mya [17, 62]. Flag appears to have been lost in uloborids and eresids, but retained in at least two RTA-clade families, Zoropsidae and Desidae (Fig. 4). The phylogenetic relationship of Pflag and Sp5803 suggests that this spidroin type was maintained in the evolution of araneoid spiders but was subsequently lost in cobweb weavers (Theridiidae); at least searches of the *Parasteatoda tepidariorum* genome and *L. hesperus* transcriptomes have failed to recover orthologs of Pflag/Sp5803 [48, 60, 82]. In the orb-web weaving spider *T. clavipes*, Sp5803 is highly expressed in flagelliform silk glands and thought to be associated with capture webs [66]. Theridiid cobwebs are highly modified relative to orb-webs, and while they have flagelliform glands, in most species glue droplets are deposited on major ampullate silk fibers, suggesting less selective pressures on genes associated with flagelliform fibers [83, 84].

Capture threads of cribellate and ecribellate spiders serve the same function, prey retention, but they achieve it using different mechanisms. Cribellate capture thread is a dry adhesive that uses a combination of hygroscopic forces, van der Waals’ forces, entanglement of cuticular structurers (e.g., setae and spines), and the absorption of cuticular waxes of prey [11, 36, 38]. By contrast, ecribellate capture thread is a wet adhesive that uses viscoelastic glue droplets composed of glycoproteins and phosphoproteins surrounded by aqueous coats of salts [84–88]. Unlike the spigots of the core axial fiber (see above), spigots associated with the production of cribellar fibrils and aqueous glue droplets in cribellate and ecribellate capture threads are not homologous [14]. In terms of amino acid repeat structure, we found no similarity in the silk genes associated with cribellar fibrils or aggregate droplets, CrSp and AgSp respectively. Moreover, phylogenetic analyses show that while CrSp and AgSp each form monophyletic clades, they are not each other’s closest relatives (Figs. 1 and 4). AgSp appears to result from a duplication event in araneoid ecribellate spiders that is independent from the origin of CrSp.

In Uloboridae, paracribellar silk connects the core axial fibers to the surrounding cribellar nanofibers [4, 8, 89]. Paracribellar spigots have been identified in some cribellate taxa and are usually located on the posterior median spinneret [35] (Fig. 4B, C). The main proteins associated with this silk type have not been identified, in part due to lack of silk-oriented studies in cribellate spider taxa. From the *U. diversus* spidroin catalog, we propose *U. div* Sp_vA and *U. div* Sp_vB as candidate genes of paracribellar silk based on their phylogenetic relationships. Spidroin types almost always form well supported monophyletic groups (e.g. all TuSp together, all AcSp together, etc.; Fig. 1; [44, 49, 57]). In contrast, *U. div* Sp_vA and *U. div* Sp_vB, failed to group with any known spidroin type, and instead both *U. div* Sp_vA and *U. div* Sp_vB clustered weakly outside of the Flag, Pflag/Sp5803, and CrSp clade (Fig. 1). To better understand the origins of *U. div* Sp_vA and *U. div* Sp_vB sequences, spidroins exclusively involved in capture thread production, including “Sp” spidroins from other cribellate species, were included in a gene tree analyses (Fig. 4). The two uloborid sequences, *U. div* Sp_vA and *U. div* Sp_vB, are positioned within a clade that includes other spidroins with unknown glandular origin (Fig. 4). Because these sequences do not share the characteristics of CrSp or Pflag spidroins and share a monophyletic origin, we propose that spidroins within *U. div* Sp_vA and *U. div* Sp_vB containing clade are likely to be expressed in paracribellate silk glands. While repetitive sequences within this clade are divergent, this could be related to a mechanism that connects the axial fiber and the cribellar fibrils to each other or a strategy for dealing with different environments. A wider sampling of cribellate spider genomes and expression studies of paracribellar glands are needed to obtain a more specific annotation of “Sp” spidroins.

Differences in the mechanical performance of spider silks have been attributed to fiber number and arrangement [33, 34], spinning rate [90], humidity [79, 91], temperature [90], and diet [92]. The molecular components of silk, especially spidroins, are also known to influence mechanical performance. The mechanical properties of cribellate capture thread have been recorded for a few species, showing it can stretch up to 500% its original length [40, 77, 93]. This extensibility is achieved because of the composite design of cribellate capture thread, with pseudoflagelliform axial fibers contributing the initial strength and extension (~ 150%) and after its breakage, the multiple cribellar fibrils unfold to allow exceptional extensibility. Ecribellate capture silk can stretch as much as 1000% when covered in aqueous glue [94, 95]. This extensibility is attributed to the presence of the tandemly arrayed amino acid motif GPG(X) in Flag that forms spring-like helices [43], with a greater number of GPG(X) motifs associated with more extensible fibers [96]. Compared to native wet araneoid silks, dry cribellate capture silks tend to have lower extensibilities and higher strengths [77, 96]. Based on these performance comparisons, it was suggested that the core fiber of cribellate capture thread spidroin (Pflag) would have similar amino acid motifs to those of ecribellate Flag spidroins, but fewer instances of proline-containing motifs, or would express different spidroin paralogs to achieve extensibility intermediate between other fiber types and araneoid wet capture silk [40]. Consistent with both hypotheses, Flag sequences indeed have a higher percentage of glycine and proline amino acids compared to Pflag sequences. Uloborid Pflag spidroin does not have the same amino acid motifs as Flag, instead, it has the motifs GPS(X), KPS(X), and QPS(X), with KPS(X) being the dominant motif in *U. diversus* Pflag (Fig. 3). These proline-containing amino acid motifs are also different from the *Deinopis spinosa* spidroin motif GPQ(X) [57].

The differences in amino acid motifs in Flag and Pflag may also partly explain differences in how axial fibers of ecribellate and cribellate capture spirals respond to water. Specifically, ecribellate flagelliform fibers are more than twice as extensible (> 100% higher) when covered in aqueous glue than when cleaned of aqueous glue [96–98]. In contrast, uloborid (cribellate) pseudoflagelliform fibers only increase extensibility by 20–30% when covered in water [97], which is more similar to proline-poor silks (i.e., minor ampullate and major ampullate silk of some species) than to proline-rich flagelliform, even though Pflag has a much higher percentage of proline than most minor ampullate spidroins [77]. The presence of glycine in the GPGX motif of Flag versus the higher prevalence of glycine-lacking motifs in uloborid Pflag may contribute to their differences in response to water and maximum extensibility. Consistent with that idea, *D. spinosa* capture threads stretch significantly more than those of Uloboridae [40], and have a higher percentage of glycine containing GPS(X) motifs in their spidroin (ABD61590) than uloborids. However, the additional undulating fibers present in *D. spinosa* cribellate capture thread [40] may be more important for its greater extensibility than abundance of GPS(X) motifs. Extensibility differences across cribellate and ecribellate capture threads are likely due to variation and abundance of specific amino acid motifs. Within cribellate species, it seems that different lineages have developed different strategies to achieve stretchiness, such as the addition of undulating fibers and the use of multiple diverse motifs in Pflag spidroins.

The overall structure of CrSp and Pflag spidroins in *U. diversus* highlights the complexity of repeat organization within these genes. Both genes are comprised of distinct repeats arranged in a stereotypical pattern. The occurrence of hierarchically organized repetitive units (i.e., ensemble repeats) has been found in other spidroins such as MaSp1 [75] and AgSp [99, 100] suggesting they may be critical to the mechanical performance of these proteins. Similarly, comparison of the *U. diversus* sequences with *Octonoba* reveals shared repetitive features that may have functional significance. For CrSp, there is clear homology between species in each of the primary repeat modules but striking differences in the overall arrangement of these modules. For Pflag, there is higher repetitive unit sequence divergence among species, but similar ensemble repeat structure. Collecting full-length CrSp and Pflag sequence for additional species within the Uloboridae will be critical to mapping the pattern of evolutionary changes in repeat organization in these genes and to understanding how these changes impact capture thread performance.

## Conclusions

Cribellate capture silk is a complex mixture of many fibers that work as a unit to adhere to prey. How these fibers are woven together affects the mechanical properties as each fiber has a distinct biophysical behavior. We described in detail the molecular components of each silk type for *U. diversus*, including a newly discovered spidroin type. For each silk type, we analyzed associated spidroins and elucidated their role in the mechanical properties of cribellate silk. Furthermore, we showed that the molecular composition of the capture thread of cribellate spiders has a complex evolutionary history involving at least six duplication events dating back to ~ 250 mya [17, 62]. While Pflag and Flag spidroins resulted from a more recent duplication and likely were co-expressed ancestrally, CrSp spidroins do not appear homologous to AgSp (do not share a recent gene duplication event) suggesting both punctuated and gradual evolution of the capture spiral. Given that cribellate threads are mainly spun by the basal members of the infraorder Araneomorphae, further knowledge of the underlying molecular composition of more primitive cribellate capture thread will surely shed light into the evolution of capture thread fibers and adhesives.

## Materials and methods

### Sample preparation and nucleic acid extractions

Mature female *U. diversus* were collected in Riverside, California, USA by T. Dugger. High molecular weight genomic DNA was extracted from a single whole individual using the Gentra PureGene tissue kit (Qiagen, Valencia, CA, USA). For RNA, cephalothorax and total silk gland tissue (a combination of all silk gland types attached to the spinnerets) were isolated from individual spiders, flash frozen in liquid nitrogen, and stored at − 80 °C. Tissues were homogenized in TRIzol reagent using a Fisherbrand bead mill 24 homogenizer (Fisher Scientific, Waltham, MA, USA). RNA was then purified using the PureLink RNA Mini kit with on-column DNAse treatment (Ambion, ThermoFisher Scientific, Wilmington, MA, USA). Because of the small size of *U. diversus*, for each RNA extraction, tissues of the same type were combined from four individuals prior to homogenization. Nucleic acid quantification was done using a Qubit Fluorometer (Thermo Fisher Scientific, Wilmington, MA, USA) and RNA integrity was assessed with a Bioanalyzer (Agilent Technologies, Santa Clara CA, USA).

### Genomic library preparation and sequencing

A total of 950 ng of high molecular weight genomic DNA extracted from a single individual was sent to the New York Genome Center (New York, NY, USA) for 10× Chromium library preparation (10X Genomics, Pleasanton, CA, USA), which included size selection (with PippinHT at 30 kb) and quality control. The library was paired-end sequenced (150 base pairs) on one lane of an Illumina HiSeqX.

### Iso-Seq library preparation and sequencing

Iso-Seq libraries were constructed from a total silk gland tissue RNA extraction following the Pacific Bioscience’s Iso-Seq protocol using the SMARTer PCR cDNA Synthesis kit (Clontech, Mountain View, CA, USA). Amplification of cDNA was done with 16 cycles for 4 reactions and 18 cycles for another four reactions. Amplification products were combined and purified using normal-phase 2× AMPure XP (Beckman Coulter, Brea, CA, USA). Cleaned cDNA was sent to the Genomics Resource Center, Institute for Genome Sciences at the University of Maryland School of Medicine (Baltimore, MD, USA), for library preparation and sequencing. In total, two SRMT cells were prepared and sequenced on a PacBio Sequel System.

### RNA-Seq library preparation and sequencing

RNA-Seq libraries were made from four RNA extractions, two from total silk gland tissue and two from cephalothorax tissue. RNA samples were sent to Novogene (Sacramento, CA, USA) for library preparation and sequenced on an Illumina HiSeq System, paired-end 150 bp.

### Transcriptome assembly and expression analyses

Raw sequencing reads were trimmed of low quality bases and adaptors using Trimmomatic [101]. Quality of filtered reads was evaluated with FastQC. All reads from cephalothorax and total silk gland tissues were combined for a *de novo* assembly transcriptome using Trinity v2.8.5 with default parameters [102]. We developed a novel Transcriptome Trimming and Annotation Pipeline (TrTAP), to select a single allele per Trinity defined gene and to remove low-confidence transcripts (Additional file 3: Figure S1). Briefly, TrTAP uses BLAST comparisons to high quality spider and arthropod genomes to identify transcripts with well-supported alignments to homologs and RSEM [103] to identify transcripts meeting a threshold of expression. Transcripts identified as chimeric using scripts from Clarke et al. 2014 or as rRNAs and tRNAs using either SILVA 18 S rRNA v123 [104] or tRNAscan [105] are excluded. The expression of representative transcripts (one per Trinity-defined gene) was obtained using RSEM v1.3.1 and each transcript was then compared to taxonomically-diverse databases such as PFAM v34.0 [106] and SwissProt v2018_03 [107] for annotation and GO term assignment. The retained transcripts are those that: (1) are the reciprocal best BLAST hits (BEST) or are the best match to a database gene, even if another allele in the Trinity-defined gene is the reciprocal best BLAST hit (2BEST); (2) are not the reciprocal best hit, but have sufficient expression and a significant BLAST hit with sufficient coverage (GOOD); or (3) have no BLASTP hit, but an ORF with sufficient length and expression (LONGORF). The rest of the transcripts are discarded. We used cutoffs of BLAST coverage of 20% of the full database gene, amino acids length of 50, and expression of 1 TPM in at least one library. Scripts and documentation for TrTAP are available at http://www.github.com/thclarke/TrTAP. Transcriptome quality was approximated with N50 and completeness evaluated by comparison to the arthropod v9 set of Universal Single-Copy Orthologs (BUSCO v 3.0.2).

Cleaned RNA-Seq reads were mapped back to the TrTAP single-copy transcriptome using Bowtie2 v2.4.2 with default parameters [108]. Differentially expressed genes were obtained from Bowtie counts of unambiguous matches using the R package DESeq2 with default parameters [109]. Genes with a Benjamini-Hochberg adjusted P-value < 0.001 were considered differentially expressed [110]. GOSlim term enrichment of the differentially expressed genes were calculated using the goseq module [111] with GO Slim terms obtained from GO Slim viewer [112] with the TrTAP assigned GO terms.

Silk gene expression was evaluated using GMAP [113] to map and align transcripts to the genome with default parameters, followed by STAR [114] which mapped RNA-seq reads to the genome, limiting the spidroin read counts to the terminal regions only. TPM values were calculated for all transcripts.

### Gene annotation and phylogenetic analyses

In addition to the TrTAP based-annotations, the genomic and Iso-Seq transcriptomic assemblies were subject to BLASTX searches (e-value < e-10) against published spider genomes: *Araneus ventricosus* (BGPR00000000.1), *Trichonephila clavipes* (MWRG00000000.1), and *Stegodyphus mimosarum* (AZAQ00000000.1), in addition to a protein database of published silk proteins downloaded from UniProtKB/SwissProt. Contigs were visually inspected and error corrected manually using Sequencher v5.4.6 and Geneious [115]. Spidroin contigs were assigned a spidroin type based on their repetitive sequence and phylogenetic relationships in the spidroin gene trees based on N- and C-terminal regions.

Selected spidroin contigs were translated, and the amino (N-) and carboxyl (C-) terminal regions were combined with spidroin sequences from published cribellate and ecribellate species (Supplementary Table S3). Alignments of the N- and C- terminal regions were done separately and then concatenated using MUSCLE implemented in Geneious (Additional file 1). Nucleotide sequences of selected AgSp, Flag, Pflag, Sp, and CrSp terminal region sequences (Additional file 3: Table S4) as well as an alignment of tandemly arrayed *U. diversus* Pflag repeat unit sequences were obtained separately using MUSCLE. Maximum likelihood analyses were constructed with 10,000 bootstrap replicates by RAxML v8 [116]. GAMMA amino acid model was used for the N- and C- concatenated and the Pflag/Flag analyses. For the Pflag repeat nucleotide alignment, sequences were clustered with the Unweighted Pair Group Method with Arithmetic Mean (UPGMA) implemented in Geneious. FigTree v1.4.4 was used to visualize the resulting trees from all analyses.

## Supplementary Information

**Additional file 1.** Protein sequence alignment of sequences used in phylogenetic analyses for Figure 1.

**Additional file 2. Table S1.** Counts and percentages of the alleles U. diversus transcriptome in each of the final TrTAP category both by total and by annotation.

**Additional file 3.** Supplementary Figures S1–S6 and Supplementary Tables S2–S4.

## Data Availability

All sequencing data used in this study are publicly available in the National Center for Biotechnology Information (NCBI) accession number PRJNA747999.
