# Supplementary material for: The evolutionary history of cribellate orb-weaver capture thread spidroins
Source: BMC Ecol Evol. 2022 Jul 9;22:89. doi: 10.1186/s12862-022-02042-5 (PMC9270836; doi:10.1186/s12862-022-02042-5)
Supplement: Supplementary file 3 — Additional file 3. Supplementary Figures S1–S6 and Supplementary Tables S2–S4. [file 12862_2022_2042_MOESM3_ESM.pdf]

Supplementary Materials

The evolutionary history of cribellate orb-weaver capture thread spidroins

Authors:

Sandra Correa-Garhwal, Richard Baker, Thomas H. Clarke, Nadia A. Ayoub, & Cheryl Y. Hayashi

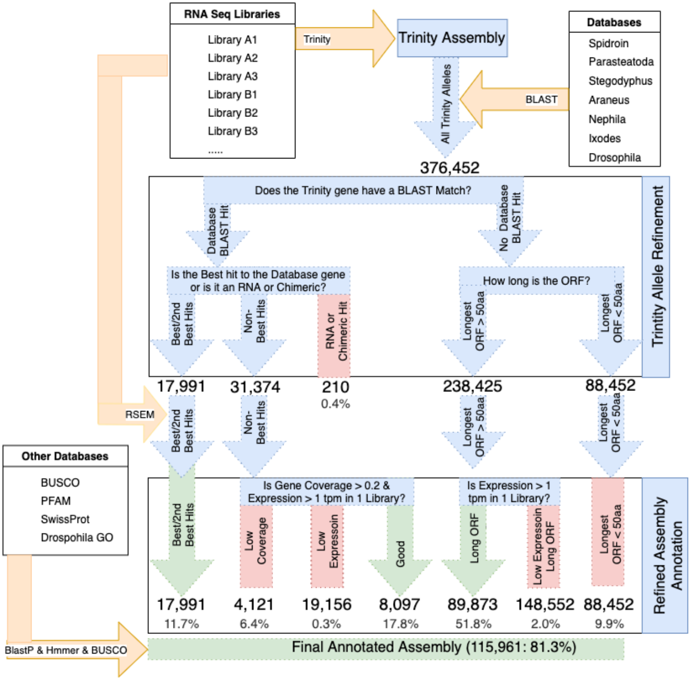

Supplementary Figure S1. Transcriptome Trimming and Annotation Pipeline (TrTAP) flowchart and results for U. diversus. The full transcriptome was run through TrTAP to identify 1 allele per Trinity-defined gene and highlight the high-confidence alleles, indicated as the final assembly. The counts of alleles in each TrTAP category at each stage are shown as the black numbers with the percentage of the summed RSEM expression across all libraries in each group are shown in dark gray numbers.

AcSp1	LSSSASSTSVVSSINAQLVPALAQTAVLNAAFSNINTQNAIRIAELLTQQVGRQYGLSGSDVATASSQIRSALYSVQQGSASTAYVSAIVGPLITVLSSRG<br>VVNASNSSQVVSSLANAILQFTANVAPQFGISIPTSAVQSDLSTISQSLTTFSSQTSSSVDSSTSAFGGISGLSGPSPYGPQPSGPSFGPGPSLSGLTGFT<br>STFASSFKSTLASSTQFQSIAQSNLDVQTRSSLISKVLINALSSLGISASVASSIAASTSQSLLSVSAGASAADVATAIAASVATSLQSSGVLTASNASQL<br>SNQLASYVSSGLSSTASSLGIQLGASLGAGFGASAGLSASTDISSSVEATSAST
TuSp1	GSQAGSQAASQAGSQSASQASASSSASAFASASAFAQSASFALSSSSSFASAVSSASSVSALGTLGYQVGLQAAGSLGISNSQAFASSISQALTSVGVGAS<br>SSAYASAVSGVVAQYLSGTGVLTSANAQALASSFANVFAASAASASAAASASSAASAQSAAAALAQSQSAASAFSQAASQAFSQAASQAGSQAASQAGSQA<br>ASQAGSQAASQAGSQAASQAGSQAASQSGSGASSFTTTISRSSAGSQTGSQAGSQAGSQAGSQAGSQAGSQAGSQAGSQAGSQAGSQAGSQA
PySp1	YQSAASTASQVSSSAAQTSSASSSMSASSFTANYNAFQSSLIQLFGSSSSFSSINTQVISASDVRNAMSSILRSSGVSGSAIQNAMSRINLSAGSSISSYA<br>QAISAAVTSAVQQSNVLSSGQEQSMASSIASQMIQTLVQLSAQRSRPPAPLPTPAPRPAPRPIPAPRPIPMPMQQPQISQLQATAAS
MiSp_vA	SSSSTQVTTQQTVTSQASAAGAGYGVGAGAGAGAGYGAGAGAGYGAGSGARAAAGAGAGYGSGAGXGAGSGYGSSAGAGAGAGAGAGAGYGAGAGAGSGAG<br>YGAGAGAGSGAGYGAGAGAGSGYGAGAGAGAGSGYGAGAGAGAGSGYGAGAGAGAGAGAGSGYGAGAGARAGAGAGTGAGYGSGYGASSGSGAGAAAGSGA<br>AAGAGYGTGAGYSTGAASAG
MiSp_C_vB	SSASAGSAINTQTVTSSTTTSSQSSAAATGAGYGTGAGTGASAGAAASGAGAGYGGQAGYGQGAGASARAAGSGYGAGAGAAAAAGSGYGTGAGAGAGSGY<br>GAGAGAGSGYGAGAGAGAGSSYGAGAGAGAGSGYGAGAAAGSGYGAGAGAGAGSGYGAGAGAGSGYGAGAGAGAGSGYGAGAGAGAGSSYSAGAGAGAGSG<br>YGAG
MiSp_N_vB	SSSSTQVTTQQTVTSQASAAGAGAGAGYGAGAGAGYGAGSGAGAASGAGYAAGAGAGAGAGYGTGAGAGAGYGAGAGAGAGAGAGYGAGAGAGSGSGYGAG<br>AGAGAGSGYGTGAGAGAGSGYGAGAGAGAGAGAGSGYGAGAGAGAVSGYGAGAGAGAGSGYSTGAGYSTGAASAG
MiSp_C_vC	SSSSTQVITQETVTSQASSGASGAASGYSAGSGAGAAAGAGAGSGYGAGAGAGAGSGYGAGAGAGAGSGYGAGAGAGAGSGYGVGAGAAAGSGYGAGAGAG<br>AGSGYGAGAGSGYGQGAGASAGGAAAGAGAGYRGQAGYGQGAGASAGAAAAGAGAGYGGQAGYGQGAGASAGAVAAGAGAGYGGQAGYGQGAGASAGAAGA<br>GAGYGGQAGYGLGAGASAGAAAAGAGAGYGGQAGYGQGAGASAGAAAAGAGA
MaSp1_N_vA	GGQTQQGGQGQGGYGSSAAAAASAAAGGLGGRGGQSGQGQGQQGYGSGASAAAAAAAGGSGGQGGQGGYGSGGQGQGGTGSGAAAAAAAAAGGSGGQGGQ
MaSp1_C_vA	AAAAAAAAGGSGGQGAQGGYGSGGQGQGQGGYGSGAASAAAAASSS
MaSp2_N_vA	GSGPGQQGPGQGPGGYGPGSAAAAAAAVGSGPGQQGPGQQGPGGYGPGSAAAAAAAAGSGPGQQGQGRQGPAGYGPGAAAAAAAGSGPGQQGPSQQGSGGY<br>GPGSAAAAA
MaSp2_C_vA	GSGPGQQGPGQGPGGYGPGSAAAAAAAAGSGPGQQGPGRQGPGGYGPGAGAAAAVAGYGPGQQGPGRQGPGGYGPGAAAAAAGSGPGPQGPGQQGPGGYGP<br>SSAAAAA
MaSp2_C_vB	GSGPGQQGPGRQGPGGYGPGAAAAAAAAGYAPGQQGPGRQGPGGYGPGAAAAAAAGSGPGQQGPGQGPGGYGPGAAAAAAAAGSGPGQQGPGQGPGVYGPS<br>SAAAAAAAA
AmSp	PQQISQYGVRQGMKPIEDSEKYYLNQHWDERNLKPNFNQEFSQPFNINQDISKDDLKVNFSPDYEADNLKLGFDHEGKVNIDFNQDLSNKVISEQNFVNQE<br>HIPRLFQEP
Sp_vA	FAAGVQAAAQVSVGAGVGVEAGVGASVDAGLDVAAAADASIGAGAAVDTSFGAAAGIGANAALSAGVSVDFSATLRTRLSAMIGATGLYSKLSSSINSAAQ<br>VKAVLLSFAGTCSRTFGVQFQSSAMNSVYSQLIQSYSARDGSFLDVLVNFASSQFVSAGILTASNLETTSNNLFRAVLTSLNASLSLGIDVDTT
Sp_vB	AVVSSNGGQSFDSGIAKSPQTSSAASSA

Supplementary Figure S2. Representative sequences of *Uloborus diversus* spidroin repetitive regions. One example of a repeat unit from each spidroin is shown. Within a spidroin, repeat units are tandem arrayed. Amino acids that are abundant in silk proteins are highlighted as follows: alanine (red), serine (purple), glycine (green), and glutamine (blue).

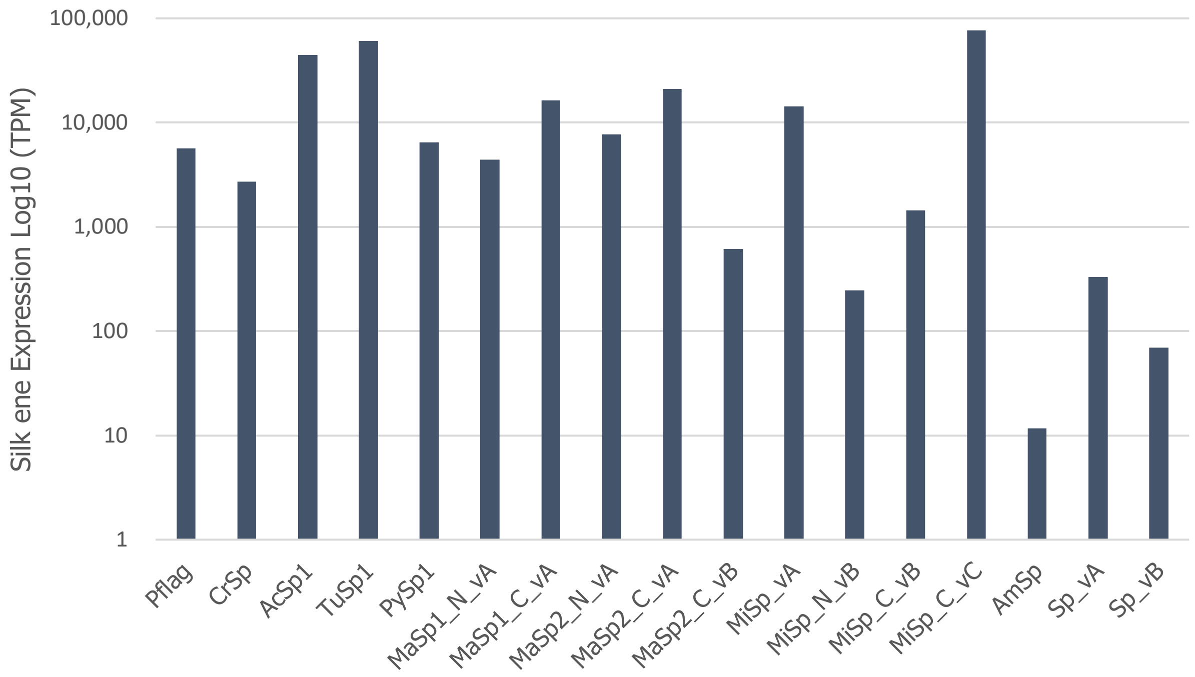

Supplementary Figure S3. Relative expression of spidroin genes in *Uloborus diversus* total silk glands. Expression shown as Log_{10} of fragment per kilobase of transcript per million (TPM).

A. Cribellar Spidroin (CrSp)

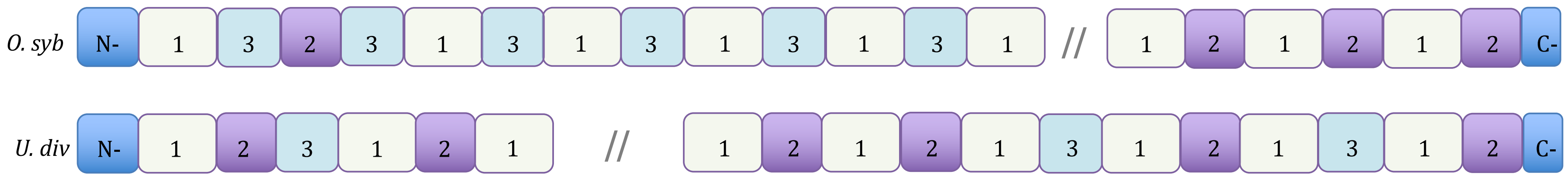

B. CrSp repeat modules

Repeat module 1

```
O.syb  PAGASAAATNLGRGIYAALASDPTFAGAFSAPFSLLRAKPVIVALARRITSFPQFGRLPLSTLVPAYINAVQKVSPGSNPAAYAQALANETGKELSANN   99
U.div  PAGASPAAANLGRGIYSALFSEPTFAGAFSAPFSLLKAKPVLVALARCITSFPQFGGLPLSALVPAYINAVQKVSPGSDPAAYAQALATTTAQALSANN   99
T.per  PAGASAVESAFGRQLYAALASNRGLSLAFSRPISLLRIRGFLSGLARYIISIRQFSSLALTDLVSTYITAVERVTLPSTITTYLQVIVEATAEILSANR   99
S.mim  RAGAPAIVSELAEQIYVALLRNPAFSLAFGGEISLETFRSFLTTFATRITSIPAFSSLLVSTLVDQNVAAVLRISAGSPLTFYAKVIAEDTAQLLYENN   99
B.lon  -AGSSDLEVAVGSHLYETLLSNPRFVSSFGLEFSLAKARVFLSALASRMHSFPQFSSLRVQDLVKRYLDALETITLGSSVSLYAQTISQVTASFLKESN   98

O.syb  LLSPEAVAAVGGQLRAAVSAALQATSQEPLAAAAAEREEGVTGERVRAGEAEAGVVEAEEAAEGEEASEEEVATEEEA----------EEAAGLEEEA-  188
U.div  LLSPQAVAAVGGELHSAVAAALQATSQEPSAAFAAEREEGVAGEGVRT-LAE-GEAEAGVG-EAEEIAESEEAAEEEEEEFEASEEDLEEAAGLEGEE-  194
T.per  LLTREAVDAASVAVRGAASSDLV---QLAEGEAGADLAAEGGAEVATATSLE-GVEEAAA---------EGVLVT-------------EEAAGRGSSPA  172
S.mim  LLTQHSLAAASSAVQTAATEATAEAEIITTTTEVAETSSAALATSETEAVPE-GTPAVSVAFAKRIYLALATDKRFVA---------------------  176
B.lon  LLSWQLISDKYEAIDEATSEA-VESIIETTPLTEKSLSTG-------LPSVE-DSAATTAATAVFSPSVLHVLSAETE---------------------  167
```

Repeat module 2

```
O.syb  ISFAKEIYNAIIGNPAFASAFSAEATLEHKHSFLNALASHITKIPEFSTVQVNELYSPYLQALKNTSTGTAF   72
U.div  LSFAKEIYNAILGNPAFSSAFSSHSTLEHKQSFLNALANHITKIPEFSTVQLNELFTSYSQALASTSTGTAL   72

O.syb  TTCARLISLTTAKVLYSKNLLTIEAFTATSAEVEEAISEALEELSAEIVAAVSIQVSSTGLPAEILKQYVT   143
U.div  STYARIIAQTTAKELYIRNLLTLEAITATSAEVEEAISEALEELSEEIVQAISTQVASVEVTAEISKPYAA   143
```

Repeat module 3

```
O.syb  AFVAPAGLSPSGVRFATTLYQQLISNAAFTSAFRVGLKKEDVIARLSGISTAFTRNERFNRMNASALTAAFTSGLGRLDAGATARRYAQLVAG    93
U.div  AFVAPAGLTPSGLRFATILYQQLLSNTAFTSAFRAGLSTQAATATLSHISTAVIRLGAFSRLPVAVLTADFNSGLGRLGVGAAASRYAQLVAG    93
T.per  TYVAPEDLSPSAADFARALYNRLLADAIFVRIFGTGLATEAAREYLSHMAIALSLVPAFKRVKPVKFTIAYRSSLSSISEGADVHAYAKGIAD    93

O.syb  CIALEASRRQLIIEGQELPLAASALSAVAAGIEAAFSTGVSAGFKEAVSGAEAGETALEATAGTEIS---------------EAAAAQAAAYVA  172
U.div  SIALEASRRQLITEGQEASLAESVVTAVSSGIEEARSAGVISGVEEVAAVAEAAETAEEITEGAEIS---------------EAAAAQAAAYVT  172
T.per  STATVLSRYGLVAEGGESDQASQVVTAFGSGIRKAEAAGVSTGTAATAAQETAVEQAAEIGATEEAGEEGIVGAEVSGVSDAELQEAQAAGYAA  187
```

Supplementary Figure S4. Comparative cribellar spidroin (CrSp) structure. **A)** Schematic of CrSp repeat region organization and its three repeat types in *Uloborus diversus* and *Octonoba sybotides*. Conserved spidroin amino and carboxyl terminal region shown as N- and C-, respectively (blue boxes). Double forward slashes indicate missing data. Numbered boxes represent repeat module types. **B**) Multiple sequence alignment of CrSp of the three repeat module types of the cribellate species *U. diversus*, *O. sybotides*, *Stegodyphus mimosarum*, *Badumna longinqua*, and *Tengella perfuga.* See **Supplementary Table S4** for sequence name information and GeneBank accession numbers. Total amino acid number shown on the right. Dashes indicate alignment gaps.

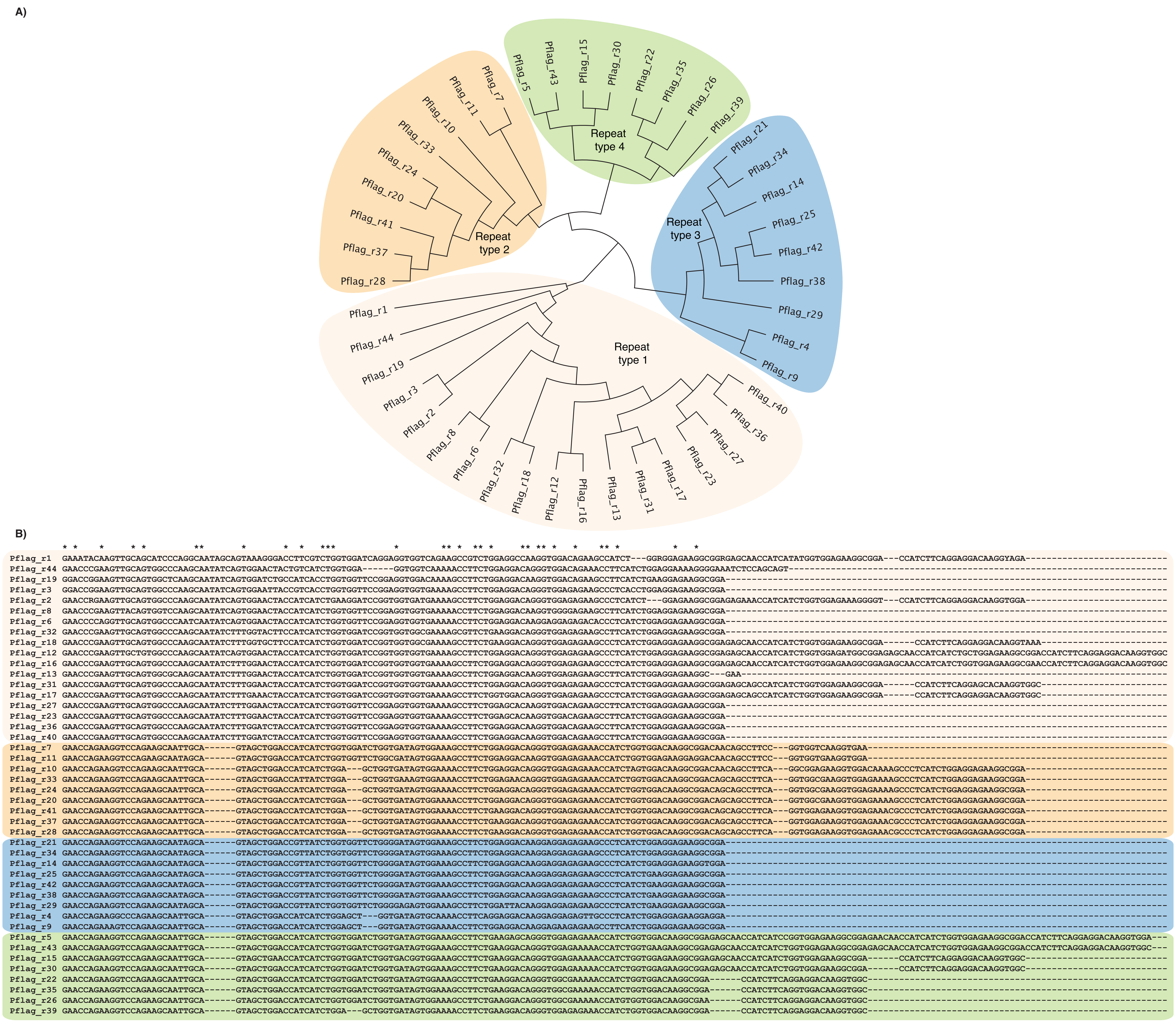

Supplementary Figure S5. A) Unrooted clustering analysis of *Uloborus diversus* Pflag repeats. B) Nucleotide alignment of Pflag repeat. Shaded shapes indicate different repeat types, cream for repeat type one, orange for repeat type two, blue for repeat type three, and green for repeat type four. Asterisk indicate nucleotides conserved across all sequences.

A.

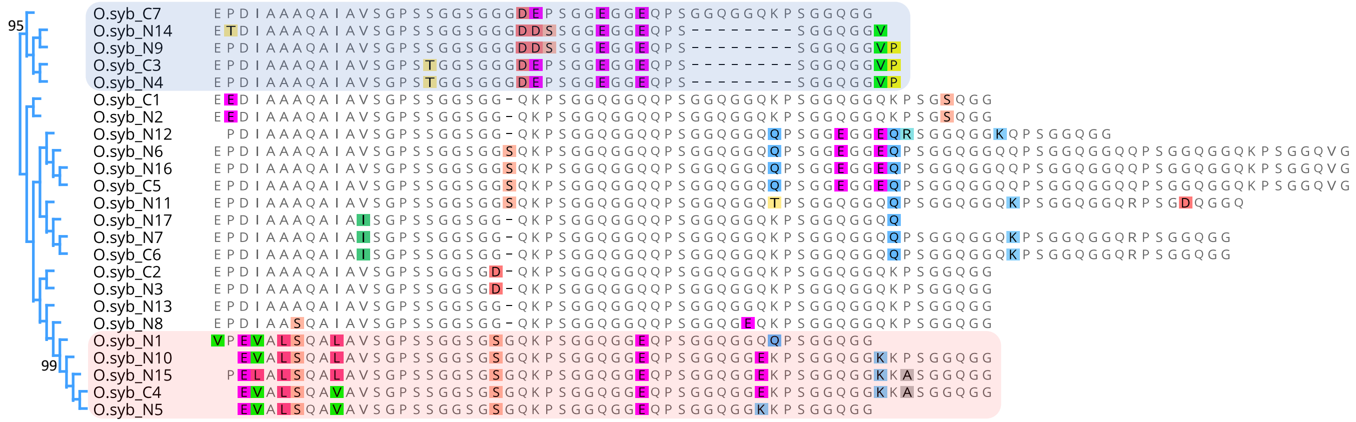

B.

```
>O.syb_N1   VPEVALSQALAVSGPSSGGSGSGQKPSGGQGGEQPSGGQGGQQPSGGQGG
>O.syb_N2   EEDIAAAQAIAVSGPSSGGSGGQKPSGGQGGQQPSGGQGGQKPSGGQGGQKPSGSQGG
>O.syb_N3   EPDIAAAQAIAVSGPSSGGSGDQKPSGGQGGQQPSGGQGGQKPSGGQGGQKPSGGQGG
>O.syb_N4   EPDIAAAQAIAVSGPSTGGSGGGDEPSGGEGGEQPSSGGQGGVP
>O.syb_N5   EVALSQAVAVSGPSSGGSGSGQKPSGGQGGEQPSGGQGGKKPSGGQGG
>O.syb_N6   EPDIAAAQAIAVSGPSSGGSGGSQKPSGGQGGQQPSGGQGGQQPSGGEGGEQPSGGQGGQQPSGGQGGQQPSGGQGGQKPSGGQVG
>O.syb_N7   EPDIAAAQAIAISGPSSGGSGGQKPSGGQGGQQPSGGQGGQKPSGGQGGQQPSGGQGGQKPSGGQGGQRPSGGQGG
>O.syb_N8   EPDIAASQAIAVSGPSSGGSGGQKPSGGQGGQQPSGGQGEQKPSGGQGGQKPSGGQGG
>O.syb_N9   EPDIAAAQAIAVSGPSSGGSGGGDDSSGGEGGEQPSSGGQGGVP
>O.syb_N10  EVALSQALAVSGPSSGGSGSGQKPSGGQGGEQPSGGQGGEKPSGGQGGKKPSGGQGG
>O.syb_N11  EPDIAAAQAIAVSGPSSGGSGGSQKPSGGQGGQQPSGGQGGQTPSGGQGGQQPSGGQGGQKPSGGQGGQRPSGDQGGQ
>O.syb_N12  PDIAAAQAIAVSGPSSGGSGGQKPSGGQGGQQPSGGQGGQQPSGGEGGEQRSGGQGGKQPSGGQGG
>O.syb_N13  EPDIAAAQAIAVSGPSSGGSGGQKPSGGQGGQQPSGGQGGQKPSGGQGGQKPSGGQGG
>O.syb_N14  ETDIAAAQAIAVSGPSSGGSGGGDDSSGGEGGEQPSSGGQGGV
>O.syb_N15  PELALSQALAVSGPSSGGSGSGQKPSGGQGGEQPSGGQGGEKPSGGQGGKKASGGQGG
>O.syb_N16  EPDIAAAQAIAVSGPSSGGSGGSQKPSGGQGGQQPSGGQGGQQPSGGEGGEQPSGGQGGQQPSGGQGGQQPSGGQGGQKPSGGQVG
>O.syb_N17  EPDIAAAQAIAISGPSSGGSGGQKPSGGQGGQQPSGGQGGQKPSGGQGGQQ

>O.syb_C1   EEDIAAAQAIAVSGPSSGGSGGQKPSGGQGGQQPSGGQGGQKPSGGQGGQKPSGSQGG
>O.syb_C2   EPDIAAAQAIAVSGPSSGGSGDQKPSGGQGGQQPSGGQGGQKPSGGQGGQKPSGGQGG
>O.syb_C3   EPDIAAAQAIAVSGPSTGGSGGGDEPSGGEGGEQPSSGGQGGVP
>O.syb_C4   EVALSQAVAVSGPSSGGSGSGQKPSGGQGGEQPSGGQGGEKPSGGQGGKKASGGQGG
>O.syb_C5   EPDIAAAQAIAVSGPSSGGSGGSQKPSGGQGGQQPSGGQGGQQPSGGEGGEQPSGGQGGQQPSGGQGGQQPSGGQGGQKPSGGQVG
>O.syb_C6   EPDIAAAQAIAISGPSSGGSGGQKPSGGQGGQQPSGGQGGQKPSGGQGGQQPSGGQGGQKPSGGQGGQRPSGGQGG
>O.syb_C7   EPDIAAAQAIAVSGPSSGGSGGGDEPSGGEGGEQPSGGQGGQKPSGGQGG
```

Supplementary Figure S6. Repeat structure of pseudoflagelliform protein in *Octonoba sybotides*. Sequence data comes from two fragments (Genbank accessions BCI65267.1 and BCI65266.1) covering the N- and C-terminal regions of the gene. Sequence names include the region (N or C) and placement of repeat unit in protein (e.g., N10 is tenth repeat unit). **A.** Alignment and phylogenetic relationships among repeat units. Variable amino acid sites highlighted by colored residues. Two strongly supported clades (red and blue) with high sequence divergence are highlighted. Bootstrap values for both nodes are provided. **B.** Organization of repeat units in *O. sybotides* protein fragments. The red and blue units correspond to sequences that belong to the red and blue clade in the alignment (**A**).

Supplementary Table S2. *Uloborus diversus* genes with homology to Theridiidae aggregate gland specific OESTs (Over Expressed Sequence Transcripts.

U. DIVERSUS GENE	TOTAL SILK (TPM)	CEPHALOTHORAX (TPM)	THERIDIIDAE CONTIG	UNIPROT HIT	ENTRY NAME	PROTEIN NAME	ORGANISM
**555378**	13.979	6.807	Lg3tf000933g1s	tr|Q5TLN1|Q5TLN1_ANGJA	Q5TLN1_ANGJA	Reverse transcriptase	*Anguilla japonica* (Japanese eel)
**5472**	24.729	8.911	Lg3tf001503g1s	tr|B7PP84|B7PP84_IXOSC	B7PP84_IXOSC	Uncharacterized protein	*Ixodes scapularis* (Black-legged tick) (Deer tick)
**556067**	3.982	7.721	Lg3tf001503g1s	tr|B7PP84|B7PP84_IXOSC	B7PP84_IXOSC	Uncharacterized protein	*Ixodes scapularis* (Black-legged tick) (Deer tick)
**407903**	11.809	139.723	Lg3tf002472g13u	tr|S7MF99|S7MF99_MYOBR	S7MF99_MYOBR	Cholinesterase	*Myotis brandtii* (Brandt's bat)
**505069**	0.000	0.950	Lg3tf002491g2u	tr|E7D162|E7D162_LATHE	E7D162_LATHE	Putative histidine decarboxylase (Fragment)	*Latrodectus hesperus* (Western black widow spider)
**568790**	2.987	8.372	Lg3tf002760g1u	tr|L7M9X6|L7M9X6_9ACAR	L7M9X6_RHIPC	Polypeptide N-acetylgalactosaminyltransferase (EC 2.4.1.-) (Protein-UDP acetylgalactosaminyltransferase)	*Rhipicephalus pulchellus* (Yellow backed tick) (*Dermacentor pulchellus*)
**2124**	12.328	14.540	Lg3tf006508g0p	tr|A8C5I4|A8C5I4_SALSA	A8C5I4_SALSA	Transposase	*Salmo salar* (Atlantic salmon)
**578025**	22.953	22.395	Lg3tf006508g0p	tr|A8C5I4|A8C5I4_SALSA	A8C5I4_SALSA	Transposase	*Salmo salar* (Atlantic salmon)
**4168**	480.703	211.455	Lg3tf011975g2p	tr|Q4PMD4|Q4PMD4_IXOSC	Q4PMD4_IXOSC	60S ribosomal protein L9	*Ixodes scapularis* (Black-legged tick) (Deer tick)
**550734**	12.264	21.716	Lh3tf000861g10p	tr|G6D1R9|G6D1R9_DANPL	G6D1R9_DANPL	Deleted.	
**698041**	12.264	21.716	Lh3tf000861g10p	tr|G6D1R9|G6D1R9_DANPL	G6D1R9_DANPL	Deleted.	
**698043**	16.327	21.174	Lh3tf000861g10p	tr|G6D1R9|G6D1R9_DANPL	G6D1R9_DANPL	Deleted.	
**569198**	36.745	68.469	Lh3tf001702g2p	tr|J9JP34|J9JP34_ACYPI	J9JP34_ACYPI	Heat shock protein 83	*Acyrthosiphon pisum* (Pea aphid)
**5597**	0.000	0.000	Lh3tf002305g4s	tr|J9JJY7|J9JJY7_ACYPI	J9JJY7_ACYPI	Uncharacterized protein	*Acyrthosiphon pisum* (Pea aphid)
**479663**	15.490	5.359	Lh3tf002305g4s	tr|J9JJY7|J9JJY7_ACYPI	J9JJY7_ACYPI	Uncharacterized protein	*Acyrthosiphon pisum* (Pea aphid)
**534154**	18.095	22.072	Lh3tf002305g4s	tr|J9JJY7|J9JJY7_ACYPI	J9JJY7_ACYPI	Uncharacterized protein	*Acyrthosiphon pisum* (Pea aphid)
**560704**	418.197	978.952	Lh3tf002305g4s	tr|J9JJY7|J9JJY7_ACYPI	J9JJY7_ACYPI	Uncharacterized protein	*Acyrthosiphon pisum* (Pea aphid)
**568850**	0.000	0.000	Lh3tf002305g4s	tr|J9JJY7|J9JJY7_ACYPI	J9JJY7_ACYPI	Uncharacterized protein	*Acyrthosiphon pisum* (Pea aphid)
**572304**	7.652	9.204	Lh3tf002305g4s	tr|J9JJY7|J9JJY7_ACYPI	J9JJY7_ACYPI	Uncharacterized protein	*Acyrthosiphon pisum* (Pea aphid)
**583445**	0.000	0.000	Lh3tf002305g4s	tr|J9JJY7|J9JJY7_ACYPI	J9JJY7_ACYPI	Uncharacterized protein	*Acyrthosiphon pisum* (Pea aphid)
**580937**	43.666	44.604	Lh3tf003562g0p	tr|J9JYX3|J9JYX3_ACYPI	J9JYX3_ACYPI	ATP synthase subunit alpha	*Acyrthosiphon pisum* (Pea aphid)

Supplementary Table S3. Spidroin sequences included in global protein phylogenetic analyses

Spidroin Name	Species	N-terminal region GenBank Accession	C-terminal region GenBank Accession
***A. arg*_AcSp**	*Argiope argentata*	AHK09813	AHK09813
***A. arg*_Flag**	*Argiope argentata*	--	MF955778
***A. arg*_MaSp1**	*Argiope argentata*	AWK58623	AWK58705
***A. arg*_MaSp2**	*Argiope argentata*	AWK58645	AWK58747
***A. arg*_MaSp3**	*Argiope argentata*	AWK58729	AWK58636
***A. arg*_MiSp**	*Argiope argentata*	AWK58671	AWK58662
***A. arg*_PySp1**	*Argiope argentata*	AQR58363	AQR58363
***A. arg*_TuSp1**	*Argiope argentata*	ATW75951	ATW75951
***A. ven*_Sp5803**	*Araneus ventricosus*	GBL75419.1	--
***B. cal*_fibroin1**	*Bothriocyrtum californicum*	HM752562	EU117162
***B. lon*_CrSp**	*Badumna longinqua*	--	GIWL01048108
***L. hes* _Flag**	*Latrodectus hesperus*	AWK58736	AWK58725
***L. hes* _MiSp**	*Latrodectus hesperus*	ARA91152	ARA91152
***L. hes*_AcSp1**	*Latrodectus hesperus*	AFX83557	AFX83557
***L. hes*_AgSp1**	*Latrodectus hesperus*	AWK58720	AMK48676
***L. hes*_MaSp1**	*Latrodectus hesperus*	F595246	F595246
***L. hes*_MaSp2**	*Latrodectus hesperus*	F595245	F595245
***L. hes*_MaSp3**	*Latrodectus hesperus*	AWK58730	AWK58638
***L. hes*_PySp1**	*Latrodectus hesperus*	AWK58659	AWK58717
***L. hes*_TuSp1**	*Latrodectus hesperus*	AWK58642	AWK58744
***O. syb*_AcSp1**	*Octonoba sybotides*	BCI65258	BCI65257
***O. syb*_CrSp**	*Octonoba sybotides*	BCI65260	BCI65259
***O. syb*_MaSp1**	*Octonoba sybotides*	BCI65263	BCI65262
***O. syb*_MaSp2**	*Octonoba sybotides*	BCI65264	BCI65264
***O. syb*_MiSp**	*Octonoba sybotides*	BCI65265	BCI65265
***O. syb*_Pflag**	*Octonoba sybotides*	BCI65267	BCI65266
***O. syb*_PySp1**	*Octonoba sybotides*	BCI65268	BCI65268
***O. syb*_TuSp1**	*Octonoba sybotides*	BCI65261	BCI65261
***S. mim*_Sp2a**	*Stegodyphus mimosarum*	KFM73910	
***S. mim*_Sp2b**	*Stegodyphus mimosarum*	KFM70693	
***T. cla*_AcSp1**	*Trichonephila clavipes*	PRD26201	PRD26201
***T. cla*_AgSp1_A**	*Trichonephila clavipes*	PRD23399	PRD23399
***T. cla*_Flag_A**	*Trichonephila clavipes*	PRD27227	PRD27227
***T. cla*_Flag_B_VeSp**	*Trichonephila clavipes*	PRD24772	PRD24772
***T. cla*_MaSp1_A**	*Trichonephila clavipes*	PRD18936	PRD18936
***T. cla*_MaSp1_B**	*Trichonephila clavipes*	PRD23750	PRD23750
***T. cla*_MaSp2_A**	*Trichonephila clavipes*	PRD23950	PRD23950
***T. cla*_MaSp2_B**	*Trichonephila clavipes*	PRD27696	PRD27696
***T. cla*_MaSp2_C**	*Trichonephila clavipes*	PRD24320	PRD24320
***T. cla*_MaSp2_D**	*Trichonephila clavipes*	PRD20448	PRD20448
***T. cla*_MaSp3_A**	*Trichonephila clavipes*	PRD19552	PRD19552
***T. cla*_MiSp_A**	*Trichonephila clavipes*	PRD23654	PRD23654
***T. cla*_MiSp_B**	*Trichonephila clavipes*	PRD30268	PRD30268
***T. cla*_MiSp_C**	*Trichonephila clavipes*	PRD24510	PRD24510
***T. cla*_MiSp_D**	*Trichonephila clavipes*	PRD18914	PRD18914
***T. cla*_PySp1**	*Trichonephila clavipes*	PRD25616	PRD25616
***T. cla*_Sp5803**	*Trichonephila clavipes*	PRD29580	--
***T. cla*_TuSp1**	*Trichonephila clavipes*	PRD35275	PRD35275

Supplementary Table S4 Spidroin nucleotide sequences of selected Flag, Pflag, Sp, and CrSp terminal region sequences included in phylogenetic analyses

Spidroin Name	Species	N-terminal region GenBank Accession	C-terminal region GenBank Accession	Full length GenBank Accession
A. arg_Flag	*Argiope argentata*	--	--	MF955787
A. tri_AgSp1	*Argiope trifascita*	--	--	MH475948
A. ven_Sp5803	*Araneus ventricosus*	BGPR01000007	--	--
B. lon_CrSp	*Badumna longinqua*		GIWL01023521	--
B. lon_Sp_NvA	*Badumna longinqua*	GIWL01038063	--	--
B. lon_Sp_NvD	*Badumna longinqua*	GIWL01010050	--	--
B. lon_Sp_Pflag	*Badumna longinqua*	GIWL01013267	--	--
D. spi_Pflag	*Deinopis spinosa*	--	DQ399325	--
L. hes_AgSp1	*Latrodectus hesperus*	MF955776	KU132352	--
L. hes_Flag	*Latrodectus hesperus*	MF955792	MF955781	--
O. gra_Pflag	*Octonoba grandiprojecta*	LC570215.	LC570214	--
O. syb_CrSp	*Octonoba sybotides*	LC570220/	LC570219	--
O. syb_Pflag	*Octonoba sybotides*	LC570227	LC570226	--
O. var_Pflag	*Octonoba varians*	LC570239	LC570238	--
O. yes_Pflag	*Octonoba yesiensis*	LC570251	LC570250	
P. tep_AgSp1	*Parasteatoda tepidariorum*	--	--	XM_021148668
P. tep_Flag	*Parasteatoda tepidariorum*	MF955795	MF955784	--
S. mim_CrSp	*Stegodyphus mimosarum*	KK117516	--	--
S. mim_Sp1	*Stegodyphus mimosarum*	--	KK113634	--
S. mim_Sp2a	*Stegodyphus mimosarum*	--	--	KK118804
S. mim_Sp2c	*Stegodyphus mimosarum*	--	--	AZAQ01099004
T. cla_AgSp1_A	*Trichonephila clavipes*	--	--	MWRG01017837
T. cla_Flag_A	*Trichonephila clavipes*	--	--	MWRG01009190
T. cla_Flag_B	*Trichonephila clavipes*	--	--	MWRG01014089
T. cla_Sp5803	*Trichonephila clavipes*	--	--	MWRG01005552
T. per_CrSp	*Tengella perfuga*	--	GGOF01121319	--
T. per_Sp_N	*Tengella perfuga*	GGOF01078382	--	--
